# Implementing care-related services in care units - an interview study

**DOI:** 10.1186/s12913-024-11465-3

**Published:** 2024-08-23

**Authors:** Dorothea Reichert, Karl Gummesson, Lars Wallin, Tobias Dahlström

**Affiliations:** 1 Centre for Clinical Research Dalarna, Uppsala University, Falun, Sweden; 2 Dalarna County Council, Falun, Sweden; 3 Department of Health and Welfare, Dalarna University, Falun, Sweden; 4 Department of Public Health and Caring Sciences, Uppsala University, Uppsala, Sweden

**Keywords:** Consolidated framework for implementation research, Qualitative content analysis, Care-related Services, Hospital Shared Services, Care unit

## Abstract

**Background:**

The growing concern about a dwindling healthcare workforce, exacerbated by demographic changes, calls for innovative solutions. One viable approach involves implementing new professional roles and restructuring existing healthcare teams within hospital care units.

**Objectives:**

To evaluate the implementation of an innovative task-shifting concept, care-related services (CRS), from the managers’ perspective in somatic care units across the hospitals in a region in Sweden.

**Methods:**

The qualitative study was conducted in 2022, after the implementation of CRS. Individual interviews were conducted with 24 key stakeholders, including 14 care unit managers, six CRS managers, and four process managers. A qualitative content analysis was performed, utilizing the Consolidated Framework of Implementation Research (CFIR).

**Results:**

The implementation of CRS involved collaboration between care unit managers, CRS managers, and project managers, alongside CRS staff, registered nurses (RNs), and licensed vocational nurses (LVNs). In particular, their roles encompassed defining boundaries, establishing routines, and managing personnel. Throughout the implementation process, challenges emerged, stemming from undefined goals, difficulties in recruiting qualified CRS staff, and issues associated with seamlessly integrating CRS into existing work routines. These challenges arose due to a constrained timeframe, widespread team apprehension, shortcomings in the training of CRS staff, unclear task allocation, and an increased workload for care unit managers. Factors associated with successful CRS implementation included effective cooperation among managers and an open-minded approach.

**Conclusions:**

Our findings highlight the crucial role of clear communication, effective recruitment, integration of CRS staff, clarification of roles, responsibilities, and defined goals for successful CRS implementation.

**Supplementary Information:**

The online version contains supplementary material available at 10.1186/s12913-024-11465-3.

## Introduction

Demographic changes have contributed towards a rising proportion of older individuals and a decreasing proportion of people in the working-age group [1]. Most notably, the population aged 80 and above, who have the highest healthcare needs, is expected to grow in Sweden as well as elsewhere in the western hemisphere [2–4]. This demographic shift will exert increased pressure on the healthcare system, leading to a heightened demand for healthcare professionals, particularly registered nurses (RNs) and licensed vocational nurses (LVNs). Consequently, this will exacerbate the current shortage of LVNs and RNs [1, 5].

Nurses’ staffing levels in care units, their training, and the patient-to-nurse ratio have been discussed and researched in a variety of ways, including in the context of nursing quality [6, 7]. Quality of care is also examined within staffing studies, focusing on the composition of staff teams and their qualifications [8]. For example, Chow and San Miguel [9] investigated nurses’ opinions regarding the implementation of assistants in nursing at hemodialysis departments, which showed a reduction in the number of clinical incidents.

In Sweden, RNs and LVNs work together in care units. While specific tasks, such as medication administration, are authorized only for RNs to perform, responsibilities are typically divided as follows: RNs are responsible for specific nursing care, documentation, and care planning, while LVNs primarily manage basic nursing care, hygiene, and food and drink provision. Care related services (CRS) can play a role in care units, as research indicates that both RNs and LVNs perform tasks that can be delegated to individuals with specific service competencies but who do not necessarily have healthcare expertise [10–12].

The implementation of CRS in hospital care units across several healthcare regions in Sweden has led to the delegation of tasks to CRS staff that were previously handled by LVNs. These tasks include cleaning of surfaces close to the patient, meal management, inventory management in the care unit, and transportation of patients [13].

Previous research has focused on how new staffing models combining RNs and nursing assistants, as well as RNs with varying qualifications and experience, have been introduced in care units [9], or on the collaboration of RNs and physicians [14, 15]. Because task shifting also helps to mitigate the shortage of skilled care personnel [16], further research on healthcare assistants' roles is needed [17, 18].

Studies conducted in Swedish regions indicate that CRS has enhanced the quality of cleaning and meal management [19–21]. At the same time, LVNs reported experiencing fewer interruptions and less stress during patient care. Moreover, patients exhibited fewer care-related infections and falls, indicating improved patient safety [20, 21]. Despite the implementation of CRS in several healthcare regions in Sweden, it remains unclear how different personnel groups collaborate and how CRS can be successfully integrated into somatic care units in hospitals.

There is a need for in-depth studies focusing on new staff models [22, 23]. Previous international research has often studied task shifting where nurses replace physicians for specific tasks [17, 18]. However, there is a lack of research regarding nursing assistants or healthcare service staff working in collaboration with RNs and research examining the implementation process of CRS.

## Aim

The present study aims to evaluate the implementation process of the innovative task shifting intervention CRS in somatic care units across the hospitals in a region in Sweden, comprehending both the prerequisites and the various factors that either facilitate or impede the implementation of CRS as described by the involved managers.

## Materials & methods

### Study design

The present study was part of a larger project aiming to increase understanding of CRS in care units and their impact on the working environment of healthcare professionals and patient care. In accordance with ethical guidelines, an ethical application was submitted (2022–03356-01), and the study design was subsequently approved by the ethics committee. Interviews with care unit managers, CRS managers, and process managers, which were conducted about six months after CRS implementation, were utilized to study the implementation process. The authors were not involved in the CRS implementation. The interview guide (see Appendix A), and the qualitative content analysis were based on the Consolidated Framework for Implementation Research (CFIR) [24]. Data analysis was validated through presentations to healthcare management and discussions with both the project manager and the research group.

### Setting

The study was conducted in a healthcare region in central Sweden. The region comprised four hospitals with approximately 26 somatic care units. In 2015, policymakers in the region made a decision to implement CRS. Subsequently, three surgery care units in hospital 1 implemented healthcare service staff in 2016. In 2019, a further decision was made to expand the implementation of CRS to an additional 17 surgery and medicine care units across all the hospitals in the region, with the primary goals being to increase the number of available beds and alleviate the workload of nursing staff. CRS were implemented in three surgery and five medicine care units at hospital 1, as well as at three surgery and three medicine care units at hospital 2 in 2021. In 2022, CRS were implemented in two care units at hospital 3 and one medicine care unit at hospital 3. At the end of 2022 all CRS had been implemented in all care units at hospital 2, 3 and 4, while some care units at hospital 1 where exempted.

### Innovation

**Fig. 1:**
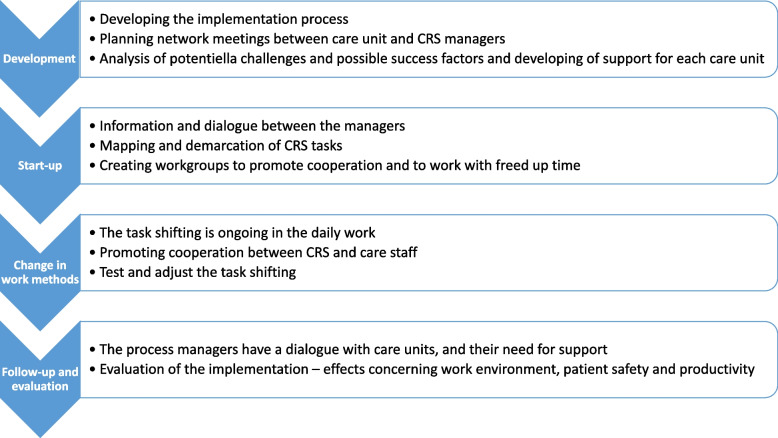
The implementation of CRS as described and used by the process managers

The implementation of CRS began with the process managers creating an implementation plan. This plan was followed by them and covered all care units described above. The plan also included the timing of the CRS implementation as well as a structure for the CRS implementation at the care units (see Fig. 1).

#### The organization of CRS

The tasks of CRS staff encompassed several areas, such as preparing patient meals with a focus on kitchen hygiene, cleaning care units including areas near patients, cleaning patient rooms after discharges, ordering and distributing consumables, and, on rare occasions, patient transport. The CRS staff was assigned to a specific care unit, which had not previously been the case with the service personnel. The costs for CRS are included in the budget for each unit. Typically, four to six CRS staff members are assigned per care unit, working closely with the nursing staff. This arrangement aims to enhance cooperation between CRS and nursing staff, allowing CRS to take on more tasks compared to for example previous cleaning services. However, care unit managers are not responsible for CRS staff or their work environment, rather CRS staff have their own managers. The CRS managers oversee one to six CRS staff teams, who work at the same clinic or hospital.

#### The implementation of CRS at the care units

The start-up phase in each care unit commenced with a dialogue and information meeting with the care unit manager, process manager, and CRS manager. Subsequently, care unit managers formed a working group with RNs and LVNs who acted as ambassadors for CRS at their care unit. The process managers had two meetings with these ambassadors to discuss their expectations and concerns about the change, cooperation with CRS staff, and the effective use of freed-up time. Each working group identified how their care unit could reorganize the division of labor by specifying and emphasizing areas and activities that RNs and LVNs wanted to allocate freed-up time to. Based on the analysis of the work tasks of nursing staff and their current job description, each care unit discussed how to use freed-up time. Some units formulated goals in connection with the implementation of CRS. For instance, one unit aimed to open all care beds, which previously had been closed due to staff shortage. Discussions also centered on defining the tasks of LVNs and RNs.

To change work methods, the focus was initially on identifying new roles and developing effective cooperation between nursing staff and CRS staff. Both employees and managers were involved in the process. CRS staff received four weeks of training, covering theory and practical aspects relating to hygiene, diet, cleaning routines, housekeeping, and patient care.

To follow up and evaluate the implementation of CRS, the process managers had a dialogue with each care unit about their further support. They also discussed effects of the CRS implementation on work environment, patient safety, and productivity.

### Participants

Care unit managers, CRS managers, and process managers were included in the study. Other managers at the hospital were excluded because they were not directly involved in the implementation of CRS but delegated the implementation of CRS to the process, care unit and CRS managers. Out of a total of 27 managers approached, 24 participated in the study. Twenty managers were female and four were male. All four approached process managers and six CRS managers participated, but only 14 of 17 care unit managers took part in the study. The care unit managers were each responsible for one care unit, while the CRS managers were responsible for one to six care units each. The process managers worked together with both CRS and care unit managers.

### Data collection

Interview data were collected in the autumn of 2022 and the spring of 2023, about six months after the start of CRS implementation. The implementation plan was used to identify all managers involved in the implementation of CRS. Participants were invited to join the study via email, and individual interview appointments were scheduled. The interviews took place in participants’ workplaces during their regular working hours. Participation in the interviews was voluntary, and participants provided written consent at the beginning of each interview. Both the first author, who is a registered nurse with a Ph.D. in nursing science, and the second author, who has a Ph.D. in industrial economics and organization, conducted the interviews. Semi-structured interview guides, based on the CFIR, were utilized. Participants were initially asked to share their experiences of and encounters with CRS and its implementation. Subsequent probing questions were adapted to the topics mentioned by the interviewees and focused on specific areas of the CFIR, such as decision-making, motivation, existing structures, management, planning, implementation, evaluation, and reflection (see Appendix A for the interview guide). The guide also included questions about the effects of the implementation, such as on patient safety and the work environment, which are not reported in this article while it focus on the implementation process. The guide did not dictate a fixed order of these areas, rather, interviews were adapted to each participant and their statements. In addition, a few in-depth questions, including inquiries about advantages, disadvantages, and concrete examples, were formulated. In order to make participation in interviews more accessible, interviewees were invited to choose the time and place for the interviews. The 24 interviews varied in duration, ranging from 11 to 71 min. All interviews were audio-recorded and later transcribed.

### Data analysis

Qualitative content analysis was conducted by applying a deductive approach [25], using CFIR codes to analyze the data (Appendix B). The analysis was performed with NVivo (QSR International, Release 1.6.1, 1137). In the first step, the first author thoroughly reviewed the interview transcripts. In the second step, meanings related to CRS and its implementation were identified and coded to the five CFIR domains. Meanings were condensed into codes, which were then grouped into CFIR domains and constructs. The research group then reviewed all the domains and constructs within the CFIR, as recommended by the CFIR founders [22]. The definition of the constructs related to the present study, summarized in the codebook, was presented after which ambiguities were discussed and the coding was reflected upon collectively. Appendix C contains the codebook consisting of the five CFIR domains and used constructs, as well as inclusion and exclusion criteria.

### Theoretical framework: CFIR

Previous research demonstrates that it is crucial for healthcare researchers to qualitatively evaluate the implementation process of innovations [22, 24, 26]. CFIR is a well-established theoretical framework in implementation research [27].

CFIR consists of five main domains, namely innovation, inner setting, outer setting, involved individuals, and the implementation process (see Appendix B) which are useful for describing and evaluating the implementation process of innovations like CRS. The innovation domain provides a detailed description of the innovation to be implemented, including evidence of strength and quality. The inner setting consists of a clear description of where the innovation is, such as a hospital. The outer setting domain refers to the external environment in which the inner setting exists, encompassing factors such as laws and organizational resources, or political aspects that influence the implementation of an innovation. The fourth domain, individuals, encompasses the motivation of the individuals involved, including leaders or officials, their roles, and characteristics. The fifth domain, the implementation process, contains in-depth knowledge of the implementation of an innovation. This part also includes understanding reflections and results [22, 24]. CFIR was initially published in 2009 and has been updated in several stages based on new studies [24].

## Results

The results of the interviews with the care unit managers, CRS managers and process managers are presented using CFIR’s five domains: Innovation, Outer Setting, Inner Setting, Individuals, and Implementation Process.

### Innovation

This category encompasses the description of the innovation as such – what the managers understanding was of CRS before implementing it. The initial testing of CRS took place in three care units at hospital 1 before it was implemented in the other care units. However, regarding relative advantages, some of the care unit managers expressed doubts about whether CRS could be effectively implemented in their care units and questioned the actual benefits it would bring. Simultaneously, the care unit managers recognized the need for CRS to increase the number of available hospital beds, improve meal-related hygiene, address the shortage of RNs and LVNs, and relieve nursing staff from tasks that can be performed by other professionals.

*”We need CRS because we cannot get enough RNs and LVNs to keep all care places open, though we need CRS.”* (Care Unit Manager)

One care unit manager mentioned that the implementation of CRS coincided with changes in their care unit that enabled better adaptation to their specific needs. The distinction between care and CRS appeared unclear to the care unit manager, as the care unit managers were less involved in the implementation process compared to CRS managers. The interviewed care unit managers expressed that they lacked sufficient knowledge about the implementation of CRS in other care units. In contrast, CRS managers claimed that they were well informed about CRS, had seen CRS in action at other hospitals, and collaborated with other CRS managers in a peer-to-peer manner.

Regarding adaptability, the respondents described both positive and negative aspects. Some managers highlighted the flexibility of work tasks and how CRS fitted the care unit requirements. Almost all care unit managers also noted the challenges brought about by rigid task descriptions for CRS and the changes required in the structure of care units to make CRS meaningful. Some care unit managers also expressed the need for more study visits at other care units to see CRS in practice and better alignment of CRS to satisfy the needs of their care units.

In addition, issues related to Innovation Complexity and Innovation Design were raised. There seemed to be confusion about the specific tasks included in CRS and the time required to complete these tasks. For example, among some of the care unit managers, it remained unclear what was included in purchased services.

*“…but I know, right now, honestly I don't have the answers to some things that I need to know, such as what exactly are the services we have purchased. What is included in these services? I personally don't know, since it specifies that cleaning for two people takes 45 minutes in a normal case. I have no idea about what is included and what we can expect apart from the fact that we have set boundaries regarding what CRS staff will do…”* (Care Unit Manager)

### Outer setting

The decision to implement CRS was primarily a result of the decision to implement CRS in all the somatic care units in the region in 2019. Some care unit managers had been advocating for the introduction of CRS for some time, while others had nothing to do with the decision. It was generally agreed that a well-thought-out plan, without haste, would be crucial to the successful implementation of CRS.

External pressure played a role in the decision-making process, particularly in terms of care quality and patient safety issues. Some care unit managers believed that faster rehabilitation, more comprehensive nursing care, and greater attention to psychological and psychosocial patient needs were necessary to maintain quality care, especially as the length of patient stays decreased. Some care unit managers expressed reducing length of stay for patients and maintaining care quality as necessary to meet the expectations of political decision-makers, and senior managers.

*“The patients should feel better, there should get safer care. We wanted to reduce the spread of infection. […] There were patient safety risks that we had difficulty getting to grips with. For example, patients were not waited on, they were not eating enough, they did not have their teeth brushed.”* (Care Unit Manager)

### Inner setting

This main category focuses on the setting where the innovation is implemented, specifically, the care unit. Respondents discussed issues related to compatibility and structural characteristics, including available resources including financing.

Concerning compatibility, some care unit managers described that the division of labor between LVNs and CRS staff made sense: LVNs are trained in care and should primarily attend to patients while CRS staff are trained in cleaning and hygiene related to food and should, therefore, primarily take on tasks related to this. Regarding common workflows, it was questionable for the care unit managers whether CRS staff had enough time for their assigned tasks. For example, a care unit manager mentioned that patients in their care unit are often discharged in the afternoon or evening when there are no CRS staff available to clean the room.

*"We have many patient discharges in the afternoon when sometimes we do not get help with all the rooms because CRS staff only work until four. And we have patients who go home perhaps at 6 pm and then we have to clean the rooms ourselves."* (Care Unit Manager)

There was often a desire to better adapt CRS to the existing structures of the care unit, both in terms of working hours and tasks. However, from the perspective of care unit management, this seemed challenging: On the one hand, there are rigid boundaries concerning task allocation, and on the other hand, CRS staff have other supervisors who determine their tasks and workflows. Therefore, making alterations or adjustments required time-consuming and challenging cooperative agreements.

In the present study, the structural characteristics are related primarily to the physical infrastructure. For example, it was claimed that there are often long distances between the kitchen and the care unit, resulting in long distances for LVNs when distributing food. Furthermore, there are not enough spaces in areas close to the patient to store the most important consumables. With regard to the compatibility of CRS, i.e. the extent to which CRS fit in with existing work processes, some participants emphasized that LVNs are relieved if CRS staff take over the daily cleaning of patient rooms and cleaning after discharge.

Funding for CRS posed challenges and uncertainties. While there was an initial project budget for implementation, subsequent financing was unclear to most care unit managers. Some were concerned that CRS staff might replace LVNs, which would result in increased personnel costs. However, it was expected that material and food costs for patients would decrease.

*"But if it were to reach a point where you have to consider replacement – meaning CRS costs – if we would have to remove LVNs’ services as a consequence, then that would be a disaster."* (Care Unit Manager)

The implementation timeline for CRS was impacted by the COVID-19 pandemic, resulting in constraints on both time and resources.

"*We were in the middle of the pandemic when we implemented CRS. We had just had a wave in the winter*, *so we were pretty stressed going into this. And that perhaps meant that we didn't have the time or resources for this.”* (Care Unit Manager)

### Individuals

Several key roles were involved in the implementation of CRS, each with distinct responsibilities: care unit managers, CRS managers and process managers.

#### Care unit managers

Care unit managers played important roles in the implementation process of CRS, even though they lacked the authority to decide independently on the implementation of CRS in their units. Their responsibilities varied at the different care units and were not always clearly defined. Care unit managers’ tasks included informing nursing staff, developing routines, and creating process descriptions, particularly concerning the supply of medical resources. Care unit managers also worked on integrating nursing staff into the implementation of CRS. After CRS had been implemented, they strived to enhance cooperation between the nursing staff and CRS staff and to improve structures and processes. Sometimes, they also took responsibility for the work environment of CRS and served as a bridge between the nursing staff, the CRS staff, and the CRS manager.

*“It is very good if CRS staff members come directly to me if something is causing problems that I can solve, so I solve it and then I feed it back to their manager.”* (Care Unit Manager)

However, care unit managers who were not involved from the beginning faced challenges due to their limited knowledge of CRS, which hindered the assignment of tasks to CRS staff.

#### CRS managers

CRS managers were responsible for defining boundaries, developing routines, and providing instructions before CRS implementation. They also organized training programs and internships for new CRS staff. During implementation, CRS managers conducted training sessions for new CRS staff and oversaw various tasks, including personnel management, fostering a positive work environment, and recruiting new CRS staff. After implementation, their responsibilities included managing sick leave among CRS staff and ensuring the allocation of tasks. Despite their pivotal role, CRS managers faced challenges due to their lack of presence on the care unit floor, which prevented them from directly observing day-to-day operations.

*“We have CRS staff who work in several care units and they have a CRS manager. But in the care unit where they work, there are other managers. And I don't have any natural insight into what it's like, because I'm not present at the care unit. I can go there and visit, but when I do they tend to stop what they are doing.”* (CRS Manager)

#### Process managers

The responsibilities for the implementation of CRS relied on existing management structures. This means that division managers, operations managers, and care unit managers were collectively responsible for the implementation. To support care unit managers and enhance cooperation between the care unit and CRS managers, a support person was hired. This individual worked closely with care unit management to promote a structured implementation of CRS. The process managers facilitated network meetings to encourage cooperation and knowledge sharing among managers, which was positively received.

### Implementation process

The implementation process of CRS can be divided into two key aspects: the implementation plan and the development of cooperation between different staff groups.

#### Implementation plan

At the beginning of the implementation, working groups were formed at the care units, focusing on task shifting, efficient use of freed-up time, and understanding the roles and responsibilities of RNs, LVNs and CRS staff respectively. However, challenges did arise due to ambiguity surrounding the specific role and disagreements about the allocation of freed-up time and its utilization. Care unit managers reflected that it was important to address these challenges by clarifying roles, defining tasks, and fostering cooperation, effective communication, and problem solving. The following quote outlines the difficulties faced by care unit managers.

*“We [the care unit managers] have carried out the procedure with some worry. We can’t escape, and maybe that’s what made it go well, that you got what it was about and what you were worried about and how it would be.”* (Care Unit Manager)

Managers emphasized that a respectable work environment for CRS staff can considerably improve their ability to support nursing staff. This included clear roles and responsibilities for CRS staff and deadlines for CRS tasks.

The implementation of CRS in care units was described as a complex process that required good motivation and task shifting methods. However, the current plan lacked a detailed and cohesive structure to effectively address the implementation challenges. The process managers described how the implementation plan lacked clear priorities, for example, decisions on which care unit would implement CRS first.

*“We [the process managers] requested priority from top management: Where should CRS be implemented first? Where do we need this the most? However, the management answered that we could handle it as we wanted. […] So, even there, it was uninteresting from the management's point of view.”* (Process Manager)

On the other hand, care unit managers faced limitations in influencing and adjusting the implementation plan. This hindered the ability to tailor the plan to better suit the unique circumstances and requirements of the specific care unit.

There were certain shortcomings in the implementation plan. The timing of the CRS implementation, particularly in the lead-up to the summer season with an increase of substitute staff due to vacations, posed challenges in effective planning and execution. The pandemic further complicated the implementation process, resulting in unexpected hurdles and delays.

The same challenges applied to the goals of the implementation. Care unit managers and CRS managers described that goals were unclear, not measurable, or absent for the whole implementation process. Some care unit managers developed their own goals for the implementation of CRS, such as reducing the duration of inpatient stays, increasing patient interaction time, minimizing care-related infections, and improving the overall patient experience.

*“We probably talked about goals, but I don't remember what they were. However, I do remember that we said we need more time with the patients to be able to spend more time in direct care.”* (Care unit manager)

#### Cooperation

Several managers emphasized the importance of developing a robust communication structure and a positive work environment for all employees. To enhance the coordination and foster a shared understanding of roles and responsibilities, some care units had regular meetings between CRS and nursing staff.

Forming a dedicated workgroup and integrating CRS into the care unit activities was described as essential in creating a welcoming atmosphere for CRS staff. However, CRS staff were not contracted to the care unit, which posed challenges, such as finding their place within the nursing team and the care unit’s routines.

*“We discuss things directly with CRS staff, they don't have to go through me but we try to get them together so that they can work together even though they have different assignments and when there are a lot of discharges I tell my staff that they can go and help the CRS staff.”* (Care Unit Manager).

The ambiguity in task allocation between nursing staff and CRS staff was a source of confusion and inefficiency during the implementation process. Misinformation had affected the effectiveness of CRS, leading to a knowledge gap that was addressed through proper training at some care units. Open dialogue and improved initial communication were something that was asked for by some care unit managers. However, both care unit managers and CRS managers described that scheduling regular meetings was challenging due to busy schedules and other priorities.

*“What is the CRS staff supposed to do and what is the nursing staff supposed to do? It can get a bit tricky. […] Then also looking further, how can we work together?”* (Care Unit Manager).

Care unit managers were not responsible for CRS staff, and they found it difficult to provide feedback and guidance. Some care unit managers were open to feedback from CRS staff, but not all felt it was necessary to engage in such discussions. Care unit managers wanted to be more involved in the recruitment process of CRS staff to ensure a good fit with their nursing team. The physical distance between CRS staff and CRS managers, and the fact that CRS staff worked at multiple care units, hindered effective communication and understanding. Addressing issues at the care unit was also complicated for the CRS managers due to this distance.

## Discussion

In summary, the decision to implement CRS, without clear goals pertaining to patient safety, work environment, or staff health, significantly influenced the entire implementation process. The perceived motive for implementing CRS varied among care unit managers because the benefits and advantages associated with CRS were not entirely clear, in addition the care unit managers were worried about the high costs of the implementation.

The fact that both RNs and LVNs perform tasks that do not require care competence [12], coupled with the shortage of RNs and LVNs [1], has led to reconsidering task shifting and professional roles within hospital care units. This has been suggested to be important for improving both staffs’ work environment and patient safety. CRS was implemented earlier in other healthcare regions in Sweden and in three other care units within the region. However, lessons from the implementation experiences in other regions were not fully utilized in the current implementation process.

It is reported by Yang et al. [28] that more assistants in care units are related to increased costs. On the other side, it has also been found that a political decision to increase the patient-to-nurse ratios presents challenges and that a sufficient budget is only one aspect of achieving safe staffing levels [29]. Some care unit managers in the present study also expressed concern about the effect on the care unit's budget of the CRS implementation.

The CRS implementation caused challenges, such as misalignments between CRS staff’s working hours and the care unit structure. As highlighted in the interviews, the implementation of CRS relied on aligning the services with existing care unit structures, including working hours and tasks. Furthermore, concerns were raised about whether CRS staff have enough time to complete their tasks. Improving the work environment for the nursing staff was only considered possible if CRS staff had adequate time for their tasks.

The implementation of CRS lacked the necessary structure, with insufficient needs analyses and limited adaption of CRS to care unit needs. Sometimes, care unit managers successfully addressed these structural challenges through their commitment. Colvin et al. describe that clear structures and well-defined processes are needed to implement a new staffing model and facilitate managers’ work [30]. Our study underscores this, as some care unit managers described that inadequate processes made the implementation of CRS difficult. CRS managers had clearly defined roles and tasks, and their responsibilities were well established. On the contrary, care unit managers were less engaged in the implementation process and their roles and tasks were less clear in comparison with CRS managers. While some care unit managers asserted responsibility for the work environment of CRS staff and collaboration between nursing staff and CRS staff, others noted that their responsibilities were not clearly defined. Furthermore, some care unit managers described facing challenges due to limited knowledge, especially as they were not involved in the implementation of CRS from the beginning. Other studies also indicate that both engagement and leadership confidence are important for successful staffing implementation, for example, knowledge about and involvement in the implementation process [31, 32].

As seen in other studies, the implementation of a new staff group presents challenges [9]. Integrating a new staff group requires effective cooperation and without active engagement and cooperation from involved groups, the implementation process is bound to be met with resistance and have setbacks. Aligning goals, accepting the innovation, and adapting the leaders’ roles among multiple leaders are crucial aspects [33]. In our study insufficient resources further exacerbated challenges that included shortages in conducting thorough analyses of the adaptations needed for existing structures and time limitations due to the ongoing pandemic.

Furthermore, nursing and CRS staff need to work together on an equal footing in order to achieve good cooperation, a positive work environment, and patient safety [16]. However, as reported in the interviews, this was not always evident. Insufficient training for CRS staff and unclear task allocation caused inefficiencies and hindered seamless integration. Role conflicts and poor teamwork have been found to be common also in other studies about skill mix [28, 34].

Our findings highlight the importance of systematically discussing both the advantages and disadvantages of organizational structures when implementing new team compositions. This is evident as the care unit managers reported having limited influence over both the activities and working hours of CRS staff, who were not integrated into the nursing staff group. Instead, CRS staff had their own managers, which created several challenges. Coordination became more complex, and changes had to be managed by different managers unfamiliar with each other's work and responsibilities. This led to delays in addressing and solving emerging needs. The decision to have separate managers for CRS staff was primarily based on the organizational separation between patient care and facility cleaning.

The scope of activities in nursing care is vast and multifaceted and involves a combination of physical, emotional, cognitive, and organizational components [35]. The implementation of CRS changed the focus of nursing staff, particularly LVNs, having to perform fewer physical tasks, especially cleaning. The organizational labor instead increased with the implementation of CRS due to the increased need for communication and team building. The increased need for time spent on coordination with CRS for RNs and LVNs can influence patient safety, work environment, and staff health in a negative way, especially since some care unit managers described that RNs were not relieved by the implementation of VNS. Care unit managers reported that LVNs had brought forward that the smoother the cooperation worked, the more nursing staff could delegate their tasks and focus on core care activities, such as nursing care, care planning, wound dressing, documentation, and patient interactions. Also Li et al. [36] has described that nonlicensed personnel can help to reduce the workload of LVNs but not of RNs on care units. RNs’ workload instead may increase due to the added burden of supervising and training LVNs and CRS staff [28].

### Strengths and limitations

Initially, the implementation of CRS was not a research project, but rather an organizational change necessitated by the shortage of RNs and LVNs. The research study was impacted by the fact that CRS were already implemented in most care units in the spring and autumn of 2021, while the study was initiated in the spring of 2022. Due to this, there was no data collection before the implementation. The planning of the implementation, the process for defining goals, and their evaluation were only studied retrospectively.

The study included care unit managers, CRS managers, and process managers, but did not involve department managers, RNs, or LVNs. The perspectives of the included managers are shaped by their managerial roles and responsibilities. However, it is important to acknowledge that the exclusion of RNs and LVNs may have limited the depth of information gained about the implementation process and cooperation within the care units. Utilizing predefined questions based on the CFIR constructs made it possible to give a structured description of the implementation of the CRS, but may have restricted the participants’ freedom to express their own opinions of the innovation. The opinions of the interviewed managers are confined to the specific timeframe of the study, given its cross-sectional design. The exclusion of the department managers may have limited the ability to analyze the impact of the outer setting, such as environmental, political or economic conditions, on the implementation of CRS. In addition, further quantitative studies are needed to increase generalizability, and additional longitudinal data are required to enhance the understanding of long-term effects.

To illuminate the implementation of CRS, we applied content analysis using the CFIR. This approach was selected to uncover the circumstances surrounding the implementation of CRS, to align our findings with other studies grounded in CFIR, and to gain a comprehensive understanding of the CRS implementation process. Utilizing the CFIR constructs helped to systematically describe and evaluate all the factors associated with the implementation. This also enhances reliability by applying what Lincoln and Guba [37] describe as theoretical triangulation in qualitative research, where an established theoretical framework serves as a methodological tool. However, the dividing line between the two CFIR domains outer setting and inner setting is not always that clear and depends largely on the specific implementation.

## Conclusion

Due to the lack of trained nursing staff, there is a necessity to explore how new staffing groups can be integrated into care units. The implementation of the non-evidence-based innovation of the new staffing group, CRS staff, requires focusing on cooperation. Creating successful cooperation between staff groups is an intrinsic part of the innovation itself, and not merely a component of the implementation process.

While certain nursing departments had long advocated for the implementation of CRS due to a chronic shortage of trained nursing staff, others with better access to trained nursing personnel perceived the implementation of CRS as less imperative. However, the hesitancy to embrace CRS stems from a dual challenge: a lack of familiarity with the innovation and skepticism regarding its overall usefulness. This skepticism manifested itself in the form of limited cooperation between CRS staff and nursing staff, coupled with a diminished commitment to the effective implementation of CRS. This lack of commitment was evident in various facets, ranging from the absence of formulated goals to ineffective communication between CRS managers and care unit managers. A motivated care unit manager is important in addressing any misgivings that nursing staff may have, establishing clear role descriptions, and fostering a positive work environment for both nursing staff and CRS staff.

### Supplementary Information

Supplementary Material 1.

## Data Availability

The datasets used during this study are available from the corresponding author on reasonable request.
