# Supplementary material for: Implementing care-related services in care units - an interview study
Source: BMC Health Serv Res. 2024 Aug 23;24:976. doi: 10.1186/s12913-024-11465-3 (PMC11344403; doi:10.1186/s12913-024-11465-3)
Supplement: Supplementary file 1 — Supplementary Material 1. [file 12913_2024_11465_MOESM1_ESM.pdf]

Implementing care-related services in care units - an interview study

Appendix A: Interview template

Introduction
- Thank you for taking the time.
- Informed consent.
- You choose yourself whether you wnat to answer the questions.
- What you convey is analyzed pseudonymised. Compiled at a level that does not single out individuals, clinics and/or hospitals.

Decisions regarding the implementation
- Who decided to introduce CRS?
- How was the decision made to introduce CRS?
- How big was the need?
- What kind of documentation did you have that showed that CRS could work in your care department?

Purpose of the study:
Analyze how the implemetation process itself succeeded and the effects of CRS.

Evaluation and Reflection
- Has CRS become part of the care process?
- Have CRS staff taken over the correct tasks?
- Collaboration between nursing and CRS staff?
- What do you value most about the introduction of CRS?
- How was the economy affected by the introduction of CRS?

Motivation
- What types of motivation exist to ensure the success of the implementation of CRS?

Patient safety
- To what extent were patients' needs considered by your organization when deciding to introduce CRS?
- Can you describe a specific story about patients' experiences with CRS?
- How essential is the introduction of CRS to meet the needs of patients? In what way does CRS contribute to meet patients' needs?

Work environment and staff health
- How do you think about the work environment when introducing CRS?
- What are your experiences regarding staff health when introducing CRS?
- Describe what you think about relieving RNs and LVNs during and after the introduction of CRS?
- How do you think CRS could make the workplace more attractive?

Describe your thoughts on the introduction of CRS in your care unit!

Implementation
- What problems/challenges did you experience in connection with the implementation of CRS?
- What types of changes or modifications have been made to make CRS work in your workplace?
- What would you do differently if CRS were to be introduced again in your care unit?

CRS tasks:
- Cleaning
- Meal management
- Textile handling
- Inventory management

Planning
- Can you describe the plan to introduce CRS?
- How was the plan to introduce CRS developed?
- What role has your implementation plan played during the implementation?
- Describe how you have followed the implementation plan.
- What steps have been taken to encourage employees to get involved in implementing CRS?
- Describe your thoughts on organizational goals?
- What goals were set?
- To what extent are set goals monitored to see if they are followed? (Feedback reports) How are the goals followed up?

CRS and existing structures
- How well does CRS fit into existing work processes?
- How has CRS been integrated into existing processes?
- What resources were there to implement and administer the introduction of CRS? Sufficient? Are there any resources or skills you lacked?

Management
- How did your organization become involved in the introduction of CRS?
- Who led the introduction of CRS? Can you describe the role of the leader?
- What is/was your role?
- Are there other people who have been supportive? tasks, role, how they affect the introduction

Support questions:
- Tell me more?
- What are the advantages/ disadvantages?
- Why/ Why not?
- Can you give an exempel?

Termination:
Is there anything in particular that you thought about before or during the interview regarding CRS?

Appendix B: CFIR

[Damschroder LJ, Reardon CM, Widerquist MAO, Lowery J. The updated Consolidated Framework for Implementation Research based on user feedback. Implementation science : IS. 2022;17(1):75]

**I. INNOVATION DOMAIN**<br>**Innovation: The "thing" being implemented, e.g., a new clinical treatment, educational program, or city service. [Document the innovation being implemented, e.g., innovation type, innovation core vs. adaptable components, using a published reporting guideline. Distinguish the innovation (the "thing" that continues when implementation is complete) from the implementation process and strategies used to implement the innovation (activities that end after implementation is complete).]**	
**Construct Name**	**Construct Definition**<br>*The degree to which:*
A. Innovation Source	The group that developed and/or visibly sponsored use of the innovation is reputable, credible, and/or trustable.
B. Innovation Evidence-Base	The innovation has robust evidence supporting its effectiveness.
C. Innovation Relative Advantage	The innovation is better than other available innovations or current practice.

D. Innovation Adaptability	The innovation can be modified, tailored, or refined to fit local context or needs.
E. Innovation Trialability	The innovation can be tested or piloted on a small scale and undone.
F. Innovation Complexity	The innovation is complicated, which may be reflected by its scope and/or the nature and number of connections and steps.
G. Innovation Design	The innovation is well designed and packaged, including how it is assembled, bundled, and presented.
H. Innovation Cost	The innovation purchase and operating costs are affordable.
**II. OUTER SETTING DOMAIN**<br>**Outer Setting: The setting in which the Inner Setting exists, e.g., hospital system, school district, state.**<br>**There may be multiple Outer Settings and/or multiple levels within the Outer Setting (e.g., community, system, state).**<br>**Project Outer Setting(s): [Document the actual Outer Setting in the project, e.g., type, location, and the boundary between the Outer Setting and the Inner Setting.]**	
**Construct Name**	**Construct Definition**<br>*The degree to which:*
A. Critical Incidents	Large-scale and/or unanticipated events disrupt implementation and/or delivery of the innovation.
B. Local Attitudes	Sociocultural values (e.g., shared responsibility in helping recipients) and beliefs (e.g., convictions about the worthiness of recipients) encourage the Outer Setting to support implementation and/or delivery of the innovation.
C. Local Conditions	Economic, environmental, political, and/or technological conditions enable the Outer Setting to support implementation and/or delivery of the innovation.
D. Partnerships & Connections	The Inner Setting is networked with external entities, including referral networks, academic affiliations, and professional organization networks.
E. Policies & Laws	Legislation, regulations, professional group guidelines and recommendations, or accreditation standards support implementation and/or delivery of the innovation.
F. Financing	Funding from external entities (e.g., grants, reimbursement) is available to implement and/or deliver the innovation.
G. External Pressure	External pressures drive implementation and/or delivery of the innovation. Note: Use this construct to capture themes related to External Pressures that are not included in the subconstructs below.
1. Societal Pressure	Mass media campaigns, advocacy groups, or social movements or protests drive implementation and/or delivery of the innovation.
2. Market Pressure	Competing with and/or imitating peer entities drives implementation and/or delivery of the innovation.
3. Performance-Measurement Pressure	Quality or benchmarking metrics or established service goals drive implementation and/or delivery of the innovation.
**III. INNER SETTING DOMAIN**<br>**Inner Setting: The setting in which the innovation is implemented, e.g., hospital, school, city. There may be multiple Inner Settings and/or multiple levels within the Inner Setting, e.g., unit, classroom, team.**<br>**Project Inner Setting(s): [Document the actual Inner Setting in the project, e.g., type, location, and the boundary between the Outer Setting and the Inner Setting.]**	
**Construct Name**	**Construct Definition**<br>*The degree to which:*
Note:	*Constructs A – D exist in the Inner Setting regardless of implementation and/or delivery of the innovation, i.e., they are persistent general characteristics of the Inner Setting.*
A. Structural Characteristics	Infrastructure components support functional performance of the Inner Setting. Note: Use this construct to capture themes related to Structural Characteristics that are not included in the subconstructs below.
1. Physical Infrastructure	Layout and configuration of space and other tangible material features support functional performance of the Inner Setting.
2. Information Technology Infrastructure	Technological systems for tele-communication, electronic documentation, and data storage, management, reporting, and analysis support functional performance of the Inner Setting.

3. Work Infrastructure	Organization of tasks and responsibilities within and between individuals and teams, and general staffing levels, support functional performance of the Inner Setting.
B. Relational Connections	There are high quality formal and informal relationships, networks, and teams within and across Inner Setting boundaries (e.g., structural, professional).
C. Communications	There are high quality formal and informal information sharing practices within and across Inner Setting boundaries (e.g., structural, professional).
D. Culture	There are shared values, beliefs, and norms across the Inner Setting. Note: Use this construct to capture themes related to Culture that are not included in the subconstructs below.
1. Human Equality-Centeredness	There are shared values, beliefs, and norms about the inherent equal worth and value of all human beings.
2. Recipient-Centeredness	There are shared values, beliefs, and norms around caring, supporting, and addressing the needs and welfare of recipients.
3. Deliverer-Centeredness	There are shared values, beliefs, and norms around caring, supporting, and addressing the needs and welfare of deliverers.
4. Learning-Centeredness	There are shared values, beliefs, and norms around psychological safety, continual improvement, and using data to inform practice.
Note:	*Constructs E – K are specific to the implementation and/or delivery of the innovation.*
E. Tension for Change	The current situation is intolerable and needs to change.
F. Compatibility	The innovation fits with workflows, systems, and processes.
G. Relative Priority	Implementing and delivering the innovation is important compared to other initiatives.
H. Incentive Systems	Tangible and/or intangible incentives and rewards and/or disincentives and punishments support implementation and delivery of the innovation.
I. Mission Alignment	Implementing and delivering the innovation is in line with the overarching commitment, purpose, or goals in the Inner Setting.
J. Available Resources	Resources are available to implement and deliver the innovation. Note: Use this construct to capture themes related to Available Resources that are not included in the subconstructs below.
1. Funding	Funding is available to implement and deliver the innovation.
2. Space	Physical space is available to implement and deliver the innovation.
3. Materials & Equipment	Supplies are available to implement and deliver the innovation.
K. Access to Knowledge & Information	Guidance and/or training is accessible to implement and deliver the innovation.
**IV. INDIVIDUALS DOMAIN**<br>**Individuals: The roles and characteristics of individuals.**	
**ROLES SUBDOMAIN**<br>**Project Roles: [Document the roles applicable to the project and their location in the Inner or Outer Setting.]**	
**Construct Name**	**Construct Definition**
A. High-level Leaders	Individuals with a high level of authority, including key decision-makers, executive leaders, or directors.
B. Mid-level Leaders	Individuals with a moderate level of authority, including leaders supervised by a high-level leader and who supervise others.
C. Opinion Leaders	Individuals with informal influence on the attitudes and behaviors of others.
D. Implementation Facilitators	Individuals with subject matter expertise who assist, coach, or support implementation.
E. Implementation Leads	Individuals who lead efforts to implement the innovation.
F. Implementation Team Members	Individuals who collaborate with and support the Implementation. Leads to implement the innovation, ideally including Innovation Deliverers and Recipients.
G. Other Implementation Support	Individuals who support the Implementation Leads and/or Implementation Team Members to implement the innovation.

H. Innovation Deliverers	Individuals who are directly or indirectly delivering the innovation.
I. Innovation Recipients	Individuals who are directly or indirectly receiving the innovation.
**CHARACTERISTICS SUBDOMAIN**<br>**Project Characteristics: [Document the characteristics applicable to the roles in the project based on the COM-B system or role-specific theories.]**	
**Construct Name**	**Construct Definition:**<br>*The degree to which:*
A. Need	The individual(s) has deficits related to survival, well-being, or personal fulfillment, which will be addressed by implementation and/or delivery of the innovation.
B. Capability	The individual(s) has interpersonal competence, knowledge, and skills to fulfill Role.
C. Opportunity	The individual(s) has availability, scope, and power to fulfill Role.
D. Motivation	The individual(s) is committed to fulfilling Role.
**V. IMPLEMENTATION PROCESS DOMAIN**<br>**Implementation Process: The activities and strategies used to implement the innovation.**<br>**Project Implementation Process: [Document the implementation process framework and/or activities and strategies being used to implement the innovation. Distinguish the implementation process used to implement the innovation (activities that end after implementation is complete) from the innovation (the "thing" that continues when implementation is complete).]**	
**Construct Name**	**Construct Definition:**<br>*The degree to which individuals:*
A. Teaming	Join together, intentionally coordinating and collaborating on interdependent tasks, to implement the innovation.
B. Assessing Needs	Collect information about priorities, preferences, and needs of people. Note: Use this construct to capture themes related to Assessing Needs that are not included in the subconstructs below.
1. Innovation Deliverers	Collect information about the priorities, preferences, and needs of deliverers to guide implementation and delivery of the innovation.
2. Innovation Recipients	Collect information about the priorities, preferences, and needs of recipients to guide implementation and delivery of the innovation.
C. Assessing Context	Collect information to identify and appraise barriers and facilitators to implementation and delivery of the innovation.
D. Planning	Identify roles and responsibilities, outline specific steps and milestones, and define goals and measures for implementation success in advance.
E. Tailoring Strategies	Choose and operationalize implementation strategies to address barriers, leverage facilitators, and fit context.
F. Engaging	Attract and encourage participation in implementation and/or the innovation. Note: Use this construct to capture themes related to Engaging that are not included in the subconstructs below.
1. Innovation Deliverers	Attract and encourage deliverers to serve on the implementation team and/or to deliver the innovation.
2. Innovation Recipients	Attract and encourage recipients to serve on the implementation team and/or participate in the innovation.
G. Doing	Implement in small steps, tests, or cycles of change to trial and cumulatively optimize delivery of the innovation.
H. Reflecting & Evaluating	Collect and discuss quantitative and qualitative information about the success of implementation. Note: Use this construct to capture themes related to Reflecting & Evaluating that are not included in the subconstructs below.
1. Implementation	Collect and discuss quantitative and qualitive information about the success of implementation.
2. Innovation	Collect and discuss quantitative and qualitative information about the success of the innovation.
I. Adapting	Modify the innovation and/or the Inner Setting for optimal fit and integration into work processes.

Appendix C: Adapted code sheet of the CFIR for the implementation of CRS.

Main categories	CFIR-Constructs	Definition	Inclusion criteria	Exclusion criteria
Innovation	Relative Advantage, Adaptability, Trialability, Complexity, Design	Description of the innovation. What is CRS before the implementation?	Include statements which describe CRS, i.e. tasks	Exclude statements related to wishes, changes
Outer setting	Policies & Laws, External pressure	Description of persons/rolls who decided that CRS will implement and how the decision communicates in the organization. Description of the outer Setting.	Include political decision and external pressure (i.e. patients' needs).	Exclude statements om persons who are involved in the implementation itself.
Inner Setting	Compatibility, Structural Characteristics, Available resources, Financing and Innovation Cost	Description of the inner setting in which the innovation is implemented (care unit).	Include description of the care unit, challenges which exist on the care unit and financing.	Exclude statements which describe person/rolls in the setting
Individuals	Leaders, Implementation Leads, Implementation Team members, Other Implementation Support	Description of the people who are involved in the implementation process.	Include rolls and their changes, opportunities and challenges.	Exclude statements that describe cooperation.
Implementation process	Planning, Doing, Adapting, Cooperation, (Teaming, Engaging, Adapting)	Description of the implementation itself. Description of teaming and engaging of individuals.	Include descriptions of the planning, i.e. steps, milestones and goals. Include descriptions of the cooperation of involved individuals.	Exclude statements that describe the setting or individuals.
